# Evaluation of Oxidative Stability of Full Fat Soybean Flour in Storage and Sensory Quality of Tuo Zaafi-Enriched with Soy Flour as Influenced by Traditional Processing Methods

**DOI:** 10.3390/foods10092192

**Published:** 2021-09-15

**Authors:** Ece Gulkirpik, Marco Toc, Richard A. Atuna, Francis K. Amagloh, Juan E. Andrade Laborde

**Affiliations:** 1 Department of Food Science and Human Nutrition, University of Illinois at Urbana-Champaign, Champaign County, IL 61801, USA; eceg2@illinois.edu (E.G.); mtoc1@illinois.edu (M.T.); 2 Department of Food Science and Technology, Nyankpala Campus, University for Development Studies, Tamale P.O. Box TL 1882, Ghana; ratuna@uds.edu.gh (R.A.A.); fkamagloh@uds.edu.gh (F.K.A.); 3 Food Science and Human Nutrition Department, University of Florida, Gainesville, FL 32611, USA

**Keywords:** soybean, full fat-soy flour, germination, roasting, oxidative stability, sensory quality

## Abstract

The oxidative stability of pretreated full-fat soybean flour (FFSF) was evaluated under commercial (Experiment I) and accelerated conditions (Experiment II). In Experiment I, soybeans were pretreated using germination, soaking (24 h), or roasting (110–120 °C), and the dried, milled FFSF was stored for 120 days under commercial storage conditions in two cities in Ghana. Acid value (AV) and peroxide value (PV) were determined. The proximate and sensory quality of Tuo Zaafi, a maize-only dish in northern Ghana enriched with 10–30% of the pretreated FFSF, was assessed. Before storage, all samples had similar PV (1.907–4.305 mEq/kg oil); however, the AV of the germinated sample was higher than that of the unprocessed samples (10.83 vs. 3.13 mgKOH/g oil; *p* < 0.001). After storage, although AV fluctuated, the PV was similar (2.39–3.74 mEq/kg oil; *p* = 1.00). Storage location showed no significant differences in terms of AV (4.96–4.97 mgKOH/g oil; *p* = 0.994), unlike PV (2.07–3.55 mEq/kg oil; *p* < 0.001). Increasing the levels of the pretreated FFSF in Tuo Zaafi resulted in lower consumer preference scores for all sensory attributes. In Experiment II, FFSF samples (dehulled and nondehulled) prepared from germination, soaking (18 h and 24 h) and roasting were evaluated under accelerated conditions (AC) of controlled temperature (45 ± 0.1 °C) and relative humidity (81 ± 1%) for AV, PV, p-anisidine value (pAV), lipoxygenase activity (LOX), color, and moisture. Pretreatment, condition, time, and their interaction affected the oxidative stability of all FFSF samples (*p* < 0.001). Roasted samples showed the highest increase in AV and pAV in both storage conditions (*p* < 0.05). Under room temperature conditions (RTC), the roasted and germinated samples had lower LOX activity (*p* < 0.05) at the end of storage time compared to that of the controls. In conclusion, germination and soaking reduced oxidation of FFSF, while roasting promotes it, despite its common use.

## 1. Introduction

Chronic undernutrition or stunting continues to afflict millions of children worldwide [1]. In 2019, over 57 million stunted children lived in Africa, the only region where the number of stunted children increased since 2000 [2]. Diets in Sub-Saharan Africa (SSA) are largely based on staple cereal or root crops. For the most part, the high stunting prevalence among children in Africa is rooted in poverty as well as the limited access and availability of high-quality protein and nutrient-dense foods [3]. The soybean is one of the most cost-effective nutrient sources and valuable agricultural products due to its ease of cultivation and high protein (~40% d.b.), fiber (~10% d.b.), and oil (~20% d.b.) contents, along with minerals and fat-soluble vitamins [4]. Moreover, soybeans and soybean products are cholesterol- and lactose-free, and unique dietary sources of isoflavones which are naturally occurring phytochemicals that were evaluated for their potential effects in ameliorating menopausal symptoms and chronic diseases [4]. Ghanaians spend approximately 40–60% of their income on food [5]. An inexpensive, nutritious protein ingredient, like soybeans, can facilitate addressing undernutrition in SSA by adding nutritive value to staple foods without altering the familiarity of the dish.

Despite its rich nutrient composition, only a small portion of soybeans produced in SSA is used directly for human consumption as products including soy flour, defatted soy flour, soy protein, soy milk, and tofu. Although full-fat soybean flour (FFSF) is easier to obtain compared to that of other soy products, the ability to use it as an ingredient is limited [6]. Due to its unsaturated fatty acid composition, FFSF products experience reduced shelf-life, especially when exposed to prevailing climatic conditions of high temperatures and relative humidity, as well as excessive sunlight [7]. Under these circumstances, development of off-flavor and the loss of essential fatty acids are expedited by the oxidative degradation of lipids during storage. Thus, lipid oxidation is a critical biochemical reaction that negatively impacts nutritional value, functionality, and sensory attributes of soybean and soybean products, as well as their storage stability, especially for full-fat or partially defatted soybean flour [7].

Traditionally, to enhance palatability and improve nutritional quality, various pretreatment methods, such as soaking, roasting, and germination were applied to cereals and legumes, including soybeans before making flour. These pretreatment methods can lead to lasting changes in the physical and biochemical composition of soybeans [8,9]. As an example, germination can improve nutrient digestibility while decreasing the concentration of some antinutritional factors in soybeans [10,11,12]. Roasting was also reported to affect color characteristics, enhance flavor, and reduce antinutrient factors in various legumes [13,14]. Soaking can influence the phytic acid content and some enzyme inhibitors by partially or fully solubilizing and eliminating them with the discarded soaking solution [9]. Although there are many studies conducted to understand the impact of these pretreatment methods on the nutritional values of legumes, there is limited evidence evaluating their effects on the oxidative stability of soybeans and soybean products.

Oxidative stability is known as the resistance of foods containing oil to oxidation during processing and storage, and it is an important parameter that indicates their quality and shelf-life [15]. In this study, acid value (AV), peroxide value (PV), p-Anisidine value (pAV), and lipoxygenase enzyme activity (LOX) were measured to understand the oxidative stability of FFSF samples. The AV measures the amount of free fatty acids in the tested sample to determine the level of lipid hydrolysis [16]. Monitoring the amount of hydroperoxides, the primary oxidation products of fats and oils during the initial oxidation phase, as a function of time can also serve as a guide for the acceptability of a food product and assess the effectiveness of a treatment on a food lipid’s stability [17]. According to O’Brien, the degree of oxidation of soybean oil in accordance with the PV can be classified in freshness (PV < 1), low oxidation (1 < PV < 5), moderate oxidation (5 < PV < 10), high oxidation (10 < PV < 20), and poor flavor (PV > 20) [18]. Additionally, the pAV measures the secondary oxidation compounds, primarily 2-alkenals and 2,4-alkadienals generated due to hydroperoxide decomposition. Aldehydes account for almost 50% of volatiles produced during lipid oxidation, and many flavor-significant aldehydes are produced from the oxidation of soybean oil [19]. Lipoxygenase (LOX, linoleate: oxygen, oxidoreductase EC 1.13.11.12) is an enzyme that catalyzes the hydroperoxidation of polyunsaturated fatty acid containing a cis,cis-1,4-pentadiene moiety and produces conjugated unsaturated fatty acid hydroperoxides in the presence of molecular oxygen [20]. Legumes seeds are known to be rich in LOX, and for soybeans, this enzyme carries a particular importance because it is one of the major contributors for formation of “greeny”, “fishy,” and “beany” off-flavors in soybeans and soybean products [21].

In this study, the effect of traditional pretreatment methods including germination (80%, form hypocotyl), soaking (18 h and 24 h), roasting (110–120 °C, 10 min), and dehulling before drying and milling on the oxidative stability of FFSF stored in ambient store conditions as of those found in Ghana was evaluated as part of Experiment I. It was followed by a consumer acceptability test and a proximate analysis of Tuo Zaafi fortified with FFSF pretreated with different methods. Tuo Zaafi is a thick cereal-based paste staple popular among the people of northern Ghana and other neighbouring countries, such as Burkina Faso, Mali, and Nigeria [22,23]. In Experiment II, the effect of pretreatment methods on oxidative stability and physical properties of FFSF were investigated under controlled storage conditions.

## 2. Materials and Methods

### 2.1. Experiment I–Ghana Field Study

A field study in Ghana investigated the effect of pretreatment methods (germination, roasting, soaking, and unprocessed) on the oxidative stability (peroxide, acid values, and free fatty acid) of FFSF under Ghanaian ambient conditions (28.31 °C and RH-75.19%) at two urban market centers, Accra and Tamale. The flours from each of the pretreatment methods were subsequently composited with white maize flour at different levels and used to prepare Tuo Zaafi (Figure S1), a popular northern Ghanaian staple for sensory evaluation.

#### 2.1.1. Pretreatment Methods

*Germination.* Soybeans were sorted and soaked in water in a 1:2 ratio for 24 h, and drained. The soaked soybeans were then transferred into a locally woven palm basket lined with jute sacks and covered completely with moist jute sacks for 2 days for germination. The germination process was terminated after 80% of the beans showed hypocotyl. The germinated soybeans were open-air dried for 3 days and then milled with a commercial miller. The full-fat-soy flour was then sifted and packaged.

*Roasting*. Sorted and cleaned soybeans were roasted (110–120 °C) in a locally fabricated toaster for 15 min. Roasted soy was cooled and then milled into flour. The flour was also allowed to cool to room temperature before it was sifted and packaged.

*Soaking*. Soybeans were soaked in water using the ratio (1:4) for 24 h. The soybeans were then drained in a plastic basket and dried in open sunlight for 2 days. The dried soybeans were then milled into a fine flour using a commercial miller.

*Raw* (*Control–untreated*). Cleaned and sorted soybeans were milled raw without applying any of the pretreatment methods to serve as a control.

#### 2.1.2. Sensory Evaluation

The “Tuo Zaafi” preparations and evaluations were carried out at the Family and Consumer Sciences Food Laboratory, University for Development Studies Nyankpala campus. Sensory studies received exempted review by the University for Development Studies Institutional Review Board because maize and soy flours are commonly consumed by the participants.

*Recruitment of participants and training*. Fifteen local women (ages 18 to 33) who had experience in Tuo Zaafi preparation were invited for the sensory assessment. The sensory evaluation process was first explained as well as the sensory attributes under evaluation. Participants gave their consents to take a part in the sensory evaluation and received compensation for their participation. The scoring system for the acceptability test (i.e., hedonic scale) was also discussed. To reduce preparation and evaluation fatigue, five of the formulations were randomly prepared daily for five days by five groups (3 women in a group). Thus, by the end of the 5 days of intervention, all 25 formulations were evaluated. On the fifth day, the top four samples that received the highest acceptability rankings were separately prepared and re-evaluated by the participants.

*Preparation of maize-soybean composite flour and Tuo Zaafi*. White maize was sourced from the open market in Tamale. The maize was cleaned, sorted, and dehulled with a commercial corn mill. The dehulled grits were then soaked in clean water in a ratio of 1:4 for 24 h. The dehulled grits were open-air dried for 24 h and then milled into fine flour. The FFSF from the various pretreatment methods was then formulated with maize flour as follows: 10%, 15%, 20%, 25%, and 30% FFSF (dry weight basis). The composite flours from various pretreatment methods were then used to prepare Tuo Zaafi for sensory evaluation. About 1 kg of coded, pretreated soy-fortified “Tuo Zaafi” flour was presented to the women groups to prepare Tuo Zaafi as it would be done at home (Figure S2) based on a common recipe. Based on the formulation a total of 25 samples of the soy fortified Tuo Zaafi were prepared and evaluated. Each day, a maximum of 3 h (9:00 am–12:00 pm) was used for the Tuo Zaafi preparation and sensory evaluation. Prepared Tuo Zaafi samples were wrapped in aluminum foil and stored in an ice-chest to maintain a uniform temperature before the sensory evaluation. Preparation and evaluation of Tuo Zaafi occurred in two separate but adjacent rooms.

*Sensory evaluation study*. After preparation, the Tuo Zaafi was allowed to cool before serving. Approximately 40 g each of the 5 randomized soy-fortified Tuo Zaafi were coded and randomly presented to each individual on plastic plates for individual evaluation. Thus, on each day of the test, all the women independently evaluated the five samples prepared as Tuo Zaafi, but did not know which were their preparations. The panelists were asked to rate the samples using as a comparison the Tuo Zaafi they eat at home as control. The sensory attributes evaluated for the formulated Tuo Zaafi included color, mouthfeel, texture, stickiness, taste, and overall acceptability using a 5-point hedonic scale (1 = dislike extremely and 5 = like extremely).

#### 2.1.3. Proximate Analysis of Soy-Enriched Tuo Zaafi

The existing 100% maize flour Tuo Zaafi and the 10% soy-enriched flour Tuo Zaafi were separately prepared, and their compositional analysis conducted in Ghana at the Food and Nutrition Analytical Laboratory, SARI. The methods described in the Official Methods of Analysis of the Association of Official Analytical Chemists (AOAC) International were used to determine the moisture (AOAC 925.10) with some slight modification by drying the samples at 105 °C overnight for approximately 12 h instead of 24 h, crude protein (AOAC 960.52), ash (923.03), and crude fat (AOAC 922.06) [24]. Total carbohydrate was computed as follows: total carbohydrate = 100 − [moisture + crude protein + total ash + crude fat]. The energy content was calculated using the Atwater factors [25].

### 2.2. Experiment II—Oxidative Stability under Accelerated Conditions Study

Two batches of FFSF samples were prepared in Ghana and shipped to the USA to be used in this study. The samples were packaged in low-density polyethylene bags, sealed and couriered to the USA in insulated boxes to minimize effect of heat and light during the traveling time. Each batch had its control sample. In the first batch (nondehulled), three different pretreatments (germination, soaking, and roasting) were applied as described previously. The FFSF samples in the second batch (dehulled) were treated in similar ways but were also dehulled. Table 1 summarizes the experimental design of the study.

The bulk samples were divided into subsamples (~25 g) and each subsample was put into 1.5 mL flat polypropylene bags (Model: S-16798, U-line Inc., Pleasant Prairie, WI, USA) and heat sealed (Model: H-1069 6″ crimper hand sealer, Uline, City, Pleasant Prairie, WI, USA). These samples were kept at two conditions: Room Temperature (RTC, 23 ± 0.1 °C, 25.7 ± 5.4% relative humidity) and Accelerated (AC, 45 ± 0.1 °C, 81 ± 1% relative humidity) conditions. The samples kept under RTC were stored in plastic containers in the dark, while the samples kept under AC were stored in an environmental chamber (Cincinnati Sub-Zero Temperature Chamber, Cincinnati Sub-Zero Products, Inc., Cincinnati, OH, USA). The relative humidity inside the environmental chamber was maintained at 81 ± 1% using saturated KCl salt solution placed inside the incubator. Conditions were monitored daily during the whole experiment. Samples were collected on 0, 4, 8, 12 weeks from both conditions.

### 2.3. Analyses of Physical Properties and Oxidative Stability Markers

In Experiment I, acid value (AV) and peroxide value (PV) were measured on day 0, 15, 50, and 120. In Experiment II, p-anisidine value (pAV), lipoxygenase activity (LOX), color, and moisture were measured in addition to AV and PV at baseline and 4, 8, and 12 weeks after. In both studies, the analyses were conducted in triplicates. Reagents used in the analyses were purchased from Sigma–Aldrich (St. Louis, MO, USA) and Fisher Scientific Co. (Montreal).

#### 2.3.1. Crude Soy Oil

The crude oil amount of FFSF samples before storage was determined as described by Yue et al. with some modifications [26]. FFSF sample (10 g) was extracted with 30 mL hexane in a centrifuge tube using a shaking incubator (Incu-Shaker Mini, Benchmark Scientific; Sayreville, NJ, USA) at 250 rpm for an hour after vortexing for about 20 s. The mixture was then centrifuged at 1663× *g* for 5 min, and the supernatant was transferred into a new tube. The extraction process was repeated one more time by adding 5 mL hexane to the precipitate, and the supernatant was collected and added to the previous one. Hexane was then evaporated from the collected supernatant by using a nitrogen evaporator (Organomation Associates; Berlin, MA, USA). The amount of oil extracted from the FFSF sample was determined by calculating the weight change in the tube holding supernatant after the solvent evaporation, and it is presented in % d.b. unit by using the moisture content of samples on day zero.

#### 2.3.2. Determination of Acid Value

The acid values of FFSF samples were determined by following AOCS Official Methods (Cd 3d-63) [27].

#### 2.3.3. Determination of Peroxide Value

The peroxide value (PV) of FFSF samples was determined according to the AOAC Official Method (Cd 8b-90) [28].

#### 2.3.4. Determination of p-Anisidine Value

pAV of FFSF samples was measured using the AOCS official method (Cd 18–90) [29].

#### 2.3.5. Determination of Lipoxygenase Enzyme Activity

LOX of FFSF samples was measured by following the initial protocol developed by Ben–Aziz et al. and as modified by on Axelrod et al. [30,31]. The LOX-1 activity of samples was measured at room temperature using a microplate reader (SpectraMax M2e, Molecular Devices; San Jose, CA, USA) using 96-well microplates. Soy extracts and substrates were added into the microplate cells at specific ratios (5 μL:295 μL or 15 μL:285 μL). The change in absorbance at 234 nm (ε = 2.5 × 10^4^ M^−1^cm^−1^) was recorded for 5 min. Residual lipoxygenase activity was calculated based on the enzymatic activity of raw soybeans (Control 1 and 2) on zero-day as 100%.

#### 2.3.6. Determination of Color Characteristics

The color characteristics (L*, a*, b*) of FFSF samples were assessed using Hunter Lab spectrocolorimeter (Labscan XE, Hunter Associates Laboratory Inc., Reston, VA, USA). Chroma (CHR) and hue angle (h°) values were calculated using the equations given in the study of Pathare et al. Total color change (∆*E*_1_ and ∆*E*_2_) values were calculated using the equations in Pathare et al.’s study with some modification [32]. While ∆*E*_1_ shows the overall color difference between a pretreated sample and control and was calculated using Equation (1), ∆*E*_2_ was estimated using Equation (2) to indicate the color change in the samples at the end of 12-week storage.

(1) $$\Delta E_{1}=\sqrt{{\left(L_{control}{}^{*}-L_{trt}{}^{*}\right)}^{2}\;+\;{\left(a_{control}{}^{*}-a_{trt}{}^{*}\right)}^{2}\;+\;{\left(b_{control}{}^{*}-b_{trt}{}^{*}\right)}^{2}}$$

(2) $$\Delta E_{2}=\sqrt{{\left(L_{t0}{}^{*}-L_{t12}{}^{*}\right)}^{2}\;+\;{\left(a_{t0}{}^{*}-a_{t12}{}^{*}\right)}^{2}\;+\;{\left(b_{t0}{}^{*}-b_{t12}{}^{*}\right)}^{2}}$$

#### 2.3.7. Determination of Moisture Content

The moisture contents (MC) of the FFSF samples were measured by a commercial moisture meter (HB 43-SE Moisture Analyzer, Mettler Toledo, Greifensee, Switzerland).

#### 2.3.8. Statistical Analysis

Data on the oxidative stability markers collected in Experiment I were subjected to Analysis of Variance (ANOVA) using the RStudio 1.3.959 software. Fisher’s least significant difference (LSD) method was used to separate means when the ANOVA results were deemed significant at *p* < 0.05.

The sensory acceptance test data were statistically analyzed using the Kruskal–Wallis nonparametric test using RStudio 1.3.959 software. The Dunn test was used to compare differences among means when the ANOVA result was significant (*p* < 0.05).

The collected data presented in the tables are the mean values ± standard deviations (SD). For Experiment II, a three-way Mixed ANOVA was conducted to evaluate the effect of pretreatment, condition, and time on physical-chemical parameters by using RStudio 1.3.959 software.

## 3. Results and Discussion

### 3.1. Experiment I—Ghana Field Study

#### 3.1.1. Oxidative Stability Markers

**Figure 1:**
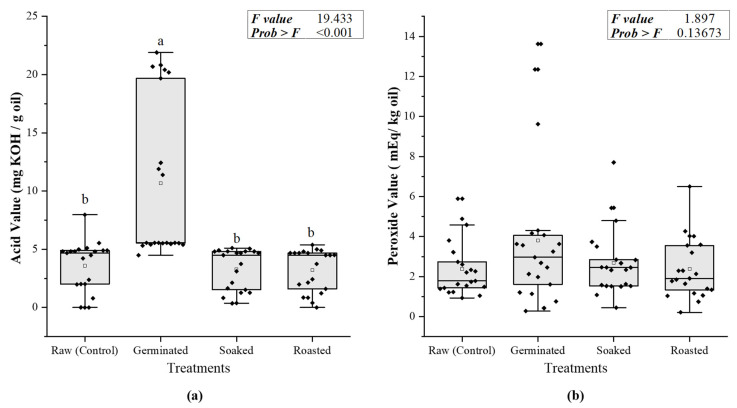
Average AV (**a**) and PV (**b**) of pretreated and raw FFSF at the end of 120 days of storage at both cities. Columns with different superscript letters (**a**,**b**) denote significant difference between treatments (*p* < 0.05).

Figure 1 shows the main effect of storage time on the average AV and PV from the two storage locations (Accra and Tamale). Germinated FFSF samples recorded a higher (*p* < 0.001) AV than other treatments. This might be due to the substantial changes in the biochemical composition of whole grains caused by germination. Soybean seed contains a high level of lipid for energy storage. During germination, triglycerides commence hydrolysis into free fatty acids (FFA), measured as AV. Although the FFA are not toxic themselves, their presence affects the sensory qualities of the product [16].

**Figure 2:**
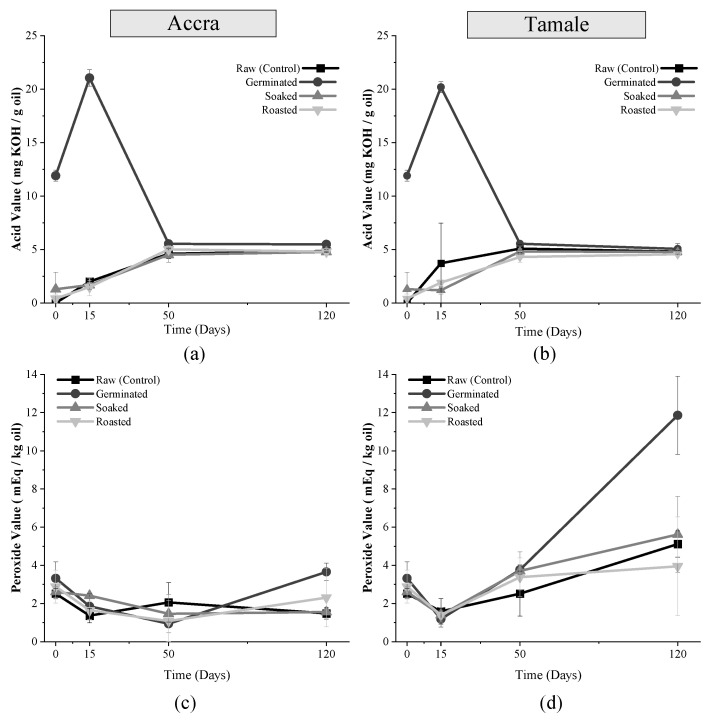
Changes in oxidative stability markers of FFSF during 120 days of storage in different locations. (**a**) AV change in samples stored in Accra; (**b**) AV change in samples stored in Tamale; (**c**) PV change in samples stored in Accra; (**d**) PV change in samples stored in Tamale.

Although germination can raise the hydroperoxide content in the seeds, there were no differences (*p* = 0.1365) in PV among all treatments [33]. Changes in AV and PV for all treatments overtime in two storage locations are shown in Figure 2. Before and after storage, the AV for all samples exceeded the CODEX- STAN 210–1999 of 0.6 mg KOH/g oil [34]. The initial increase in the acid value could be ascribed to the fact that the area of contact with oxygen is increased and the heat generated during milling of soybeans [34]. The decline in PV of samples in both location after 50 days of storage could be due to the breakdown of the primary oxidation products like hydroperoxides into secondary oxidation products like aldehydes. Generally, in the two storage locations, the AV of FFSF samples increased after 50 days of storage and levelled off subsequently, except for the germinated FFSF samples, which had a higher initial AV (*p* < 0.001) and presented an increase in the first 15 days of storage followed by a sharp decrease until the 50th day. This finding lends support to previous studies by Park et al., who reported an increase in AV of FFSF samples after 4 weeks of storage and a subsequent decline [35]. The stable trend in the AV of FFSF samples after 50 days of storage in this current study could be due to stable conditions (temperature) that resulted in equal production of free fatty acids and their subsequent decomposition into secondary oxidation products. Earlier work showed that primary oxidative product like free fatty acids, measured by AV becomes stable at room temperature and in the absence of metals [36]. Moreover, the primary oxidative products formed at the initial stages of oxidation decompose into low-molecular weight oxygenated constituents, during the final stages leading to the formation of secondary oxidative product like alcohol, aldehydes, and ketones [36,37].

Most samples’ peroxide values were below the limit set by CODEX-STAN 210–1999 (10 mEq/kg) for oils [34]. In general, the PV declined after 50 and 15 days of storage in Accra and Tamale, respectively, and increased thereafter. The initial high peroxide values observed in the two locations could be because the samples were already oxidized during the milling process. Prolonged exposure of samples to oxygen and intense heat during milling of soybean into FFSF was reported to influence the initial increase in hydroperoxides in FFSF [35]. The increase in the PV of samples after the decline could be attributed to the oxidation of residual FFA in the FFSF amid prolonged exposure to oxygen, and high humidity (75.19%) and temperature (28.31 °C) conditions.

Germinated samples had a higher AV compared to that of the other pretreated samples stored in the two locations. Substantial changes in the biochemical composition of whole grains during germination possibly accounted for this observation. Germination can increase the enzymatic activity of the endogenous lipases, which hydrolyze fats and oils into FFA [38]. This can cause a distinctive increase in the AV of the germinated samples. The generated free unsaturated fatty acids can be oxidized into hydroperoxides leading to the slightly increased PV in the germinated FFSF samples.

#### 3.1.2. Sensory Study

The results for the sensory evaluation are presented in Figures S3–S8. All the six sensory attributes assessed (i.e., color, mouthfeel, texture, stickiness, taste, and overall acceptability) showed differences (*p* < 0.001) based on the proportion of soy flour incorporated. For the most part, an increase in soy flour above 20% resulted in lower Tuo Zaafi acceptability. The inclusion of legumes to cereal-based staples in order to improve their nutrient densities was previously suggested and encouraged [39].

The color of a food product is the first attribute that influences consumers’ decision when purchasing and/or consuming it [40]. Generally, participants’ ratings for the attribute color decreased as the incorporation level of soy flour increased for all the pretreatment methods except for the germinated and the control samples (Figure S3). Beyond 20% soy flour incorporation, participants either were indifferent or disliked the color of Tuo Zaafi prepared from all pretreatment methods. Most consumers in SSA are familiar with white maize, the main ingredient in Tuo Zaafi [41]. This could explain why there was a gradual low acceptability score for color as the increased incorporation soy flour darkened the flour. Soy flour from the soaked pretreatment had a stable score of up to 25% incorporation level and then declined. This is not surprising because flour from the treatment looked whitish unlike the golden-brown color of roasted samples after milling.

Mouthfeel refers to the tactile aspects of texture perception during consumption [42]. In this study, consumers’ ratings for the soy enriched Tuo Zaafi concerning mouthfeel generally declined with increased levels of soy flour incorporation (Figure S4). The data suggested that soy flour could be composited with white maize flour up to 20% in the preparation of Tuo Zaafi for all the pretreatment methods except for the germinated and roasted pretreatment methods. This could be due to substantial changes in the lipid content. For instance, the presence and level of fat in a product affect the sensorial properties including the mouthfeel [43]. Earlier studies showed that as germination progresses in soybeans, the levels of lipid decreased because they served as the main carbon source for seed growth [44].

Texture is very useful in defining the culinary quality of foods and could have a great impact on food consumption [45]. The sensory scores for Tuo Zaafi prepared from composited flours from raw, roasted, and soaked pretreatment methods had a stable sensory score as the incorporation of soy flour increased up to 20%. Beyond this, consumers’ likeness decreased except for the roasted treatment (Figure S5).

Stickiness is described as the force required to separate the fingers after compressing the Tuo Zaafi between the thumb and forefinger. Stickiness is an important sensory attribute for Tuo Zaafi and most Ghanaian staples as they are mostly starch-based. A well-cooked Tuo Zaafi should not stick between the thumb and the forefinger but must easily be molded. As presented in Figure S6, consumers’ rating for stickiness was highest at 15% inclusion levels of soy flour for all the processing methods except for the roasted and soaked samples that were had higher acceptability up to 25% inclusion. The roasted and soaked flours incorporation possibly increased the amylose and amylopectin content of the flours since they were reported to be mainly responsible for the stickiness of food products [46].

In general, consumers liked the taste of Tuo Zaafi fortified with 10–15% soy flour for all the processing methods investigated except for the Tuo Zaafi with the unprocessed soy flour (Figure S7). Beyond 15% incorporation level, consumers were either indifferent to the additional amount of soy flour or disliked the taste of the Tuo Zaafi prepared with soy flour from all pretreatment methods.

Overall acceptability is an important sensory attribute that represents the sum of all the sensory attributes as judged by the consumers. To a large extent, overall acceptability indicates whether a product would be acceptable to consumers or not. Tuo Zaafi prepared with soy flour initially soaked scored the highest (*p* < 0.001) based on the proportion of soy flour incorporated. Addition of soy flour beyond 20% reduced the overall acceptability of all treatments (Figure S8).

Traditionally, the soaking of grains or legumes before milling into flour is a common practice in Ghana, and as such, it would be expected to be not only feasible but perhaps more culturally acceptable and effectively promoted than other techniques.

#### 3.1.3. Proximate Analysis of Soy-Enriched Tuo Zaafi

Enrichment of Tuo Zaafi with 10% FFSF resulted in a significant increase in the protein (*p* = 0.001; 12.75 vs. 7.89 g/100 g), total ash (*p* < 0.001; 1.41 vs. 0.41 g/100 g), crude fat (*p* < 0.001; 1.40 vs. 0.36 g/100 g), and total energy (*p* < 0.001; 387.54 vs. 382.87 g/kcal) content compared to that of the 100% maize flour Tuo Zaafi (Table 2). The protein, total ash, crude fat, and energy increased in the 10% soy-enriched Tuo Zaafi almost 1.6-fold, 3.4-fold, 3.9-fold, and 1-fold, respectively, compared to that of the 100% maize-based Tuo Zaafi. Similar trends were reported by Ikya et al., in whose study FFSF was composited with maize flour in the preparation of traditional Nigerian staple similar to Tuo Zaafi [25]. The protein, total ash, and crude fat increased as expected because soy is reported to contain significant amount of quality protein, lipids, and minerals [47,48]. Total carbohydrate content of the soy-enriched Tuo Zaafi was significantly (*p* < 0.001) lower than that of the maize-based control. Compared to that of FFSF, white maize flour contains 3–4-times the amount of total carbohydrates, most of which is starch [49]. Thus, the data corroborate previous studies showing a lower presence of carbohydrates as inclusion of soy flour increases [25,50].

### 3.2. Experiment II—Oxidative Stability under Accelerated Conditions Study

**Figure 3:**
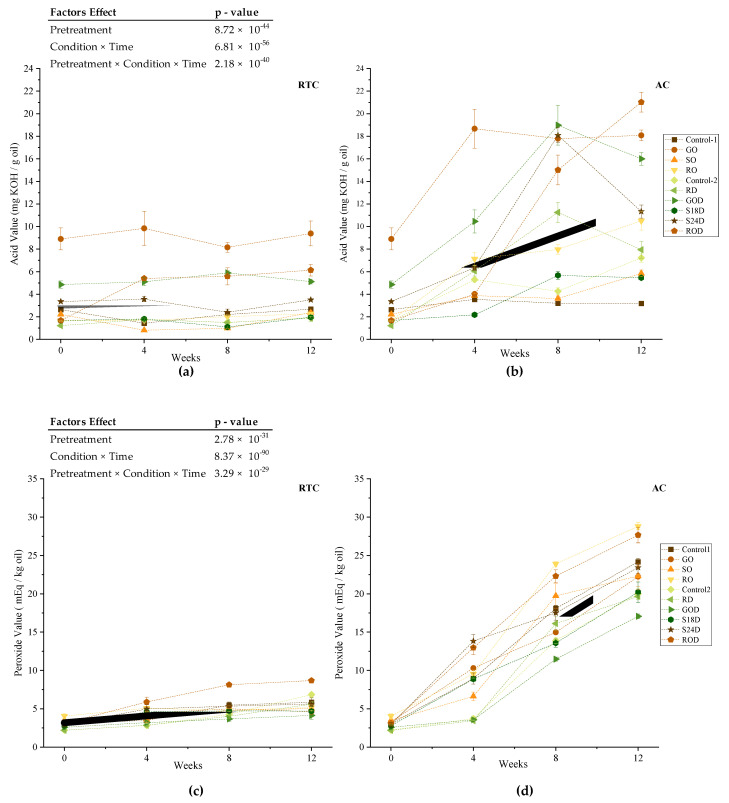
Changes in oxidative stability markers of FFSF kept under RTC and AC during 12-week storage. Thick gray lines represent overall pretreatment trend effect within each condition. (**a**) Change in AV under RTC; (**b**) change in AV under AC; (**c**) change in PV under RTC; (**d**) change in PV under AC. (Control-1 FFSF = control sample of non-dehulled batch; germinated only FFSF = GO; soaked only FFSF = SO; roasted only = RO; Control-2 FFSF = control samples of dehulled batch; raw dehulled FFSF = RD; germinated and dehulled FFSF = GOD; soaked 18 h and dehulled FFSF = S18D; soaked 24 h and dehulled = S24 h; roasted and dehulled = ROD).

**Figure 4:**
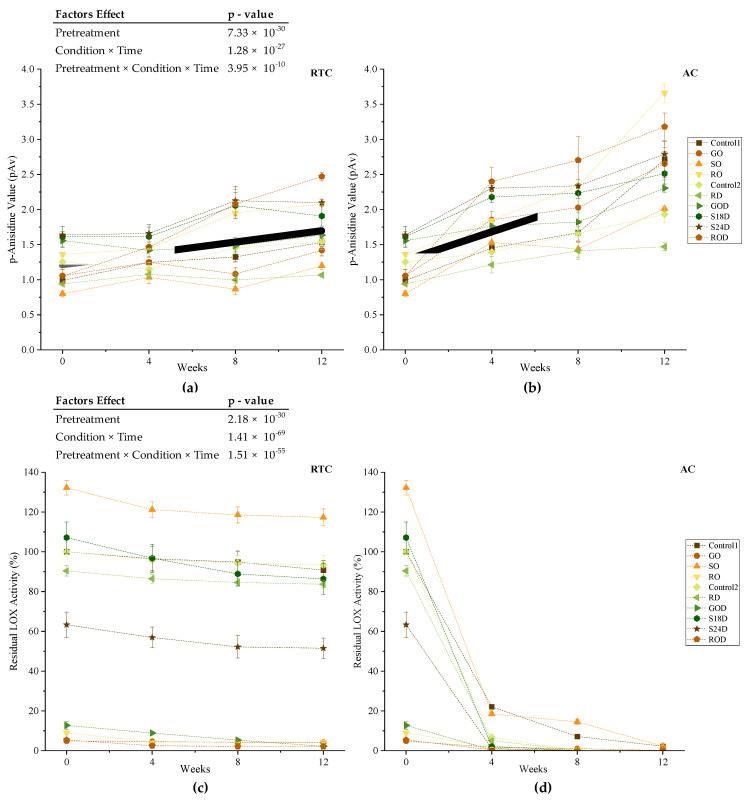
Changes in oxidative stability markers of FFSF kept under RTC and AC during 12-week storage. Thick gray lines represent overall pretreatment trend effect within each condition (**a**) Change in pAV under RTC; (**b**) change in pAV under AC; (**c**) change in residual LOX activity under RTC; (**d**) change in residual LOX activity under AC. (Control-1 FFSF = control sample of non-dehulled batch; germinated only FFSF = GO; soaked only FFSF = SO; roasted only = RO; Control-2 FFSF = control samples of dehulled batch; raw dehulled FFSF = RD; germinated and dehulled FFSF = GOD; soaked 18 h and dehulled FFSF = S18D; soaked 24 h and dehulled = S24 h; roasted and dehulled = ROD).

Figure 3 and Figure 4 show the oxidative stability markers data of FFSF samples throughout 12-week storage under RTC and AC. As shown in these figures, the three-way mixed ANOVA results showed that pretreatment method, time × condition, and pretreatment × time × condition had a significant effect on all oxidative stability markers (*p* < 0.0001). In general, the AV, PV, and pAV of FFSF samples showed a gradual increase with time in both conditions. The increase in these parameters was more pronounced under AC regardless of the pretreatment methods.

#### 3.2.1. Acid Value

The initial amount of free fatty acids formed through lipid hydrolysis, the AV, of FFSF were in the range of 1.21 mg KOH/g–8.90 mg KOH/g. Control groups of two FFSF batches were found to be significantly different in terms of their initial acid values (*p* = 0.0013). In general, the FFSF samples from the second batch showed a higher change in AV, including the Control-2 sample, than that of the samples belonging to the 1st batch. This might be due to the initial total fat content of soybeans, which may be different between batches as the grains were purchased from the open market. As shown in Figure S9, the second batch control had higher oil content (22.31 ± 0.37% vs. 21.62 ± 0.17% d.b., *p* = 0.041) than the first batch control.

As observed in Experiment I, GO samples presented the highest AV (8.90 mg KOH/g) compared to all other treatments (*p* < 0.05). These values were followed by those from the GD and S24D samples with 4.86 mg KOH/g and 3.36 mg KOH/g, respectively. There were no differences found between Control- 2, RD, RO, ROD, and S18D samples (*p* > 0.05) on zero (0) day. In agreeement with the data from the Ghana field study, AV exceeded the CODEX limit before storage conditions [34].

Exposing the samples to high temperature and humidity conditions resulted in an increase in AV for all treatments. Lipid oxidation rate can be accelerated at high temperatures, and this condition was used to exacerbate any potential differences between flour pretreatments [51]. Roasting soybeans resulted in the highest increase in AV under both conditions compared to that of the controls: from 1.68 mg KOH/g to 6.13 mg KOH/g under RTC and to 21.02 mg KOH/g under AC. Similar results were observed in the study of Park et al., in which changes in the oxidation markers (AV and PV) of raw and roasted soybean flour were monitored for 48 weeks under refrigeration, room-, and high-temperature conditions [35]. In another study, roasting soybeans at 100 °C for 20 min and 40 min both caused an increase in free fatty acid concentration and AV compared to that of raw samples (*p* < 0.05) [52]. Altogether these results suggest roasting predisposes soy flour for hydrolysis and temperature accelerates this reaction.

Under RTC conditions, the AV of all FFSF samples showed a slight increment but the change in AV of Control-1 after 12 weeks of storage. On the other hand, under AC condition, GO sample showed the second least change in AV (8.90 mg KOH/g to 18.08 mg KOH/g) during storage, which was not significantly different from the Control-1’s increase (*p* = 0.3195).

#### 3.2.2. Peroxide Value

Overall, the PV increased over time in both conditions (Figure 3c,d). However, the AC caused more increase in PV than RTC as expected. Based on O’Brien’s classification, the samples at RT are between low-oxidation and moderate-oxidation after 12 weeks of storage [18]. Whereas at AC, the samples were classified as high-oxidation and poor flavor after 12 weeks of storage. In general, the highest PV was observed in the roasted treatments (*p* < 0.05). Conversely, germinated showed low PV. Regarding soaked treatments (i.e., SO, S18D, S24D), they showed similar but intermediate-to-low PV values compared to that of the other treatments (*p* < 0.05). Soaked soybeans can result in higher lipid oxidation compared to that of unsoaked soybeans [53]. However, soaking is a milder treatment than roasting. Hydroperoxides are relatively stable at room temperature but are readily degraded by metals, high temperature, oxygen, and light into secondary products such as aldehydes, ketones, epoxides, hydroxy compounds, oligomers, and polymers [54]. Therefore, other methods such as p-anisidine value are suggested to complement the measurement of lipid oxidation.

#### 3.2.3. p-Anisidine Value

In general, pAV increased over time in both conditions (*p* <0.05), but AC had an accentuated increase (Figure 4a,b). After 12 weeks, the samples under AC showed a steeper increase of pAV compared to that of the samples at RTC. Overall, the highest pAV was observed in roasted treatments after 12 weeks (*p* < 0.05) in both conditions. High temperatures accelerate lipid oxidation [55]. In contrast, soaked (i.e., SO, S18D and S24D) and germinated samples showed intermediate to low pAV throughout the storage in both conditions. This can be attributed to the process of soaking and germination, which involves complex physical and metabolic changes such as the hydrolysis of the seed’s components due to water and oxygen, which can result in the reduction of lipid oxidation [56,57,58].

#### 3.2.4. Lipoxygenase Enzyme Activity

The RTC was more instrumental in offering more insights as to the effects of pretreatment methods on LOX activity. As shown in the Figure 4c,d, pretreatment methods, time and condition factors and their interactions reduced the LOX activity. Under AC, the LOX activity for all treatments was almost zero after four weeks of storage. Similarly, Park et al. found that LOX activity in untreated soy flour samples stored at high temperature conditions showed a significant reduction over time, and there was a significant difference between untreated and roasted samples constantly [35].

At baseline, roasted and germinated samples had lower LOX activities than other samples throughout the storage time (*p* < 0.05) under either condition. As previously shown, thermal exposure inactivates LOX enzyme [57,59,60,61]. Roasting of soybeans is a commonly employed dry heat processing method to improve the flavor, color, and nutrient digestibility, and reduce antinutritional factors in seeds [14,62]. Kermasha et al. reported that inactivation of soybean lipoxygenase can be achieved in the temperature range between 60 and 90 °C [59]. High-temperature application during the roasting process might cause denaturation and inactivation of the LOX enzyme. As shown by Park et al., roasting soy flour reduces LOX as compared with that of untreated control [35]. In another study, roasting soybeans for 20 min at 110 °C decreased the LOX activity by almost 50% [60]. Germination also reportedly might have a reductive impact on soybean LOX enzyme. In the study of Kumar et al., two different soybean genotypes were germinated at 25 and 35 °C, and the activities of three LOX isoenzymes were investigated from 0 to 144 h of germination [61]. By the end of 48 h of germination at both temperatures, the LOX-1 activities of both genotypes decreased more than 20%. Despite varietal differences that influence germination effects, this technique is associated with decreased LOX-1 activity [63,64,65,66]. Moreover, Wang et al. found that lipoxygenases were not involved in lipid mobilization in germinating seeds [67]. It is possible that instead of their known enzymatic action, they might be functioning as storage proteins during germination [67,68]. Another possible reason for the reduced LOX activity in germinated soybeans could be that they might be degraded during germination [61].

Prior to the storage, SO (132.17%) and S18D (107.15%) samples started with a significantly higher LOX activity than that of the control samples (*p* = 5.834 × 10^−10^ and *p* = 0.0218, respectively). One of the conditions required for LOX to be activated is the presence of water. With soaking, the LOX enzyme can be activated in the whole soybeans and then can be released from the kernel by grinding and they act upon substrates available in the matrix [69].

Dehulling resulted in lower LOX activities. There was a significant difference between Control-2 vs. RD (*p* = 3.66 × 10^−3^), SO vs. S24D (*p* = 4.37 × 10^−16^), and SO vs. S18D (*p* = 3.83 × 10^−8^) samples in terms of their initial enzyme activities. Dehulling can facilitate removal of some antinutrient compounds and deteriorative enzymes from legume seeds, including enzyme inhibitors, peroxidase, tannins, phytic acid, and oligosaccharides [70,71,72,73].

The reduction observed in the enzyme activity during storage under RTC might be due to the change in pH in the samples. As shown in Table S1, the moisture contents of all FFSF samples significantly increased during the storage time under RTC. The increase in moisture content might promote the formation of free fatty acid in the samples, leading to slight increment in AV, as earlier mentioned. This might then lead to an increase in the acidity of samples and inhibit the activity of LOX enzyme. LOX-1 isoenzyme acts on polyunsaturated fatty acids most effective at pH 9.0–9.5. In the study of Asbi et al., the maximum activity of LOX-1 isoenzyme was recorded at pH 9.2 [74]. Below this pH level, a sharp decrease in the enzyme activity observed until pH 5.6, and at pH levels lower than 5.6, the activity of LOX-1 was too low to be measured. In another study, it was also highlighted that LOX-1 had 3% of its activity at pH 6.8 compared to that of pH 9.0 [31].

#### 3.2.5. Color Values

L*, a*, b*, chroma and hue angle values of FFSF samples are shown in Tables S2–S7. According to the mixed ANOVA results, pretreatment method, time × condition, and pretreatment × time × condition had a significant effect on color values (*p* < 0.0001).

**Figure 5:**
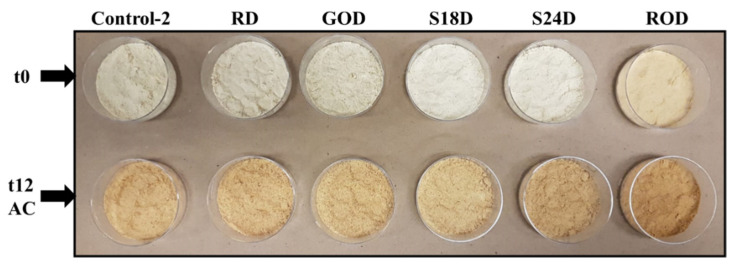
Change in color of FFSF samples kept under AC for 12-weeks (t12) compared to day zero (t0).

Changes in L* values were more notorious during the storage of flours (Figure 5). On day zero, the L* value of FFSF samples was in the range of 69.56 ± 0.44–84.98 ± 0.38. In general, under both conditions, all flour samples darkened (i.e., lower L* values), a trend that was more pronounced under AC (ranging from 51.66 ± 0.22 to 71.47 ± 0.29 on week 12) than under RTC (ranging from 67.31 ± 0.24 to 81.26 ±0.26 on week 12). Interestingly, both germinated samples (GO and GOD) were significantly darker (*p* = 1.94 × 10^−18^ and *p* = 5.91 × 10^−6^, respectively) than their corresponding controls. According to Chimna et al., the reduction in L* value in sprouted grains can be attributed to biochemical changes that take place during germination process [75]. A reduction in L* values in germinated mung bean flour, as well as a decreasing trend with increased germination time, were reported [76]. It was suggested that lower ash content or changes in protein and starch compositions in the germinated seeds might cause a decrease in L* values [76]. Roasted soybeans (RO and ROD) resulted in lower L* values than their corresponding controls, as well (*p* = 2.86 × 10^−6^ and *p* = 1.13 × 10^−12^, respectively). Similarly, Shin et al. reported that soy flour made from soybeans roasted at 140 °C for 30 min presented a significantly lower L* value than that of control, germinated, and steamed soy flour samples [65]. The change in color values, especially the L* value, during heat treatments, like roasting, might occur because of the pigment degradation, oxidation of polyphenols, Maillard reactions, and caramelization process [77].

Although there was not a clear trend in a* and b* values under RTC condition, both significantly increased under AC by the end of storage time (*p* < 0.05). A similar trend in a* value of soybeans stored at different relative humidity and temperature conditions was also observed in the studies of Yousif [78] and Kong et al. [79]. Both studies concluded that increasing the MC and/or relative humidity as well as the temperature during storage results in an increase in a* values of soybeans. Compared to that of the controls, RO and ROD samples had higher a* and b* values $(p={1.18\times 10}^{-12}\;,\;p={2.95\times 10}^{-27},\;\mathrm{respectively})$ on day zero (t_0_). In another study, roasted soy flour had significantly higher a* and b* values than nontreated soy flour (*p* < 0.05), and these differences were attributed to the effect of high temperature application during the roasting process [65].

The changes in a* and b* also affected the chroma and hue angle values of FFSF samples (Tables S6 and S7). As shown in Figure 5, samples became darker and more brownish compared to those at day zero, which was also supported by the increase in chroma and the decrease in hue angle values.

Table S7 shows the total color change (Δ*E*_1_) between control and pretreated samples on day zero (t0) and the total color change (Δ*E*_2_) in samples kept under RTC and AC at the end of the 12-week storage. According to Pathare et al., when ∆*E* is between 1.5 and 3, panelists can notice a color difference; if ∆E is higher than 3, panelists can detect two different colors directly [32]. Based on Δ*E*_1_, there was a visible total color change caused by all pretreatment methods. The highest Δ*E*_1_ was recorded in the GO samples, and it was significantly different than GOD ($p<{9.68\;\times \;10}^{-16}$), which might be due to the difference between their germination degree and the effect of dehulling. Total color change (Δ*E*_2_) was higher in FFSF samples stored under AC than under RTC. Controls 1 and 2 had the lowest Δ*E*_2_ values (15.1 ± 1.5 and 16.4 ± 0.2, respectively), whereas the highest Δ*E*_2_ values were observed in GO and ROD samples, with Δ*E*_2_ values of 29.3 ± 0.6 and 23.0 ± 0.22, respectively (Table S8).

## 4. Conclusions

The Ghana field study and accelerated conditions study showed that pretreatment, storage condition, and time alone and together have a defined impact on the oxidative stability of FFSF. Though not optimized in this study, traditional processing methods such as germination and soaking improve oxidative stability, and therefore, expand the shelf life of FFSF. Despite its common use and recommendation, roasting promotes oxidation under both accelerated and normal storage conditions. The study also illustrated that Tuo Zaafi prepared from 10% FFSF pretreated with traditional methods, such as soaking and germination, and composited with polished white maize flour is a significant source of quality protein and energy, and is acceptable to Ghanaian consumers.

## Figures and Tables

**Table 1:** Summary of experimental design of the oxidative stability under accelerated conditions study.

Batch ID		Pretreatment Methods	Sample Codes	Storage Conditions	Sample Collection
1	Dehulled	Raw (untreated)	Control-1	Room Temperature Conditions (RTC) Accelerated Conditions (AC)	Day zero Week 4 Week 8 Week 12
		Germinated	GO		
		Soaked	SO		
		Roasted	RO		
		Raw (untreated)	Control-2		
2	Nondehulled	Raw and dehulled	RD		
		Germinated and dehulled	GOD		
		Soaked 18 h and dehulled	S18D		
		Soaked 24 h and dehulled	S24D		
		Roasted and dehulled	ROD		

**Table 2:** Proximate composition and caloric value of 10% soy-enriched vs. 100% maize flour Tuo Zaafi.

Tuo Zaafi	Proximate (g/100 g)					Total Energy kcal/100 g
	Moisture	Crude Protein	Total Ash	Crude Fat	Total CHO	
10% Soy-enriched	81.72 ± 0.04	12.75 ± 0.64	1.41 ± 0.09	1.40 ± 0.09	84.44 ± 0.52	387.54 ± 0.52
100% Maize	82.80 ± 0.08	7.89 ± 0.36	0.41 ± 0.07	0.36 ± 0.06	91.33 ± 0.30	382.87 ± 1.55
*p*-value	<0.001	<0.001	<0.001	<0.001	<0.001	0.008

Values are means ± SD; n = 3. Except for moisture all values are on dry matter basis.

## Data Availability

The datasets generated and/or analyzed during the current study are available from the corresponding author on reasonable request.

## Supplementary Materials

The following are available online at https://www.mdpi.com/article/10.3390/foods10092192/s1, Table S1: initial and final moisture contents of soy samples kept under RTC and AC, Table S2: L* values of FFSF samples at day zero, week 4 (t4), week 8 (t8), and week 12 (t12) under RTC and AC, Table S3: a* values of FFSF samples at day zero, week 4 (t4), week 8 (t8), and week 12 (t12) under RTC and AC, Table S4: b* values of FFSF samples at day zero, week 4 (t4), week 8 (t8), and week 12 (t12) under RT and AC, Table S5: Chroma values of FFSF samples at day zero, week 4 (t4), week 8 (t8), and week 12 (t12) under RTC and AC, Table S6: hue angle values of FFSF samples at day zero, week 4 (t4), week 8 (t8), and week 12 (t12) under RT and AC, Table S7: Total color change (Δ*E*_1_) between control and pretreated samples on day zero (t0) and total color change (Δ*E*_2_) in samples kept under RTC and AC at the end of 12-week storage time, Figure S1: flow chart for the preparation of 1.5 kg Tuo Zaafi, Figure S2: preparation of Tuo Zaafi by women for sensory evaluation, Figure S3: sensory score for color of soy-enriched (n = 15), Figure S4: Sensory score for mouthfeel of soy-enriched (n = 15), Figure S5: sensory score for texture of soy-enriched (n = 15), Figure S6: sensory score for stickiness of soy-enriched (n = 15), Figure S7: sensory score for taste of soy-enriched (n = 15), Figure S8: sensory score for overall acceptability of soy-enriched (n = 15), Figure S9: crude oil yields of FFSF samples measured on day zero prior to the storage.
