# Supplementary material for: Evaluation of Oxidative Stability of Full Fat Soybean Flour in Storage and Sensory Quality of Tuo Zaafi-Enriched with Soy Flour as Influenced by Traditional Processing Methods
Source: Foods. 2021 Sep 15;10(9):2192. doi: 10.3390/foods10092192 (PMC8472710; doi:10.3390/foods10092192)
Supplement: Supplementary file 1 [file foods-10-02192-s001.zip › foods-1353473-supplementary.pdf]

Supplementary Materials

Table S1. Initial and final moisture contents of soy samples kept under RTC and AC.

Pretreatment	Initial MC (%)	Final MC (%) RTC		Final MC (%) AC	
Control_1	5.14 ± 0.05	5.56 ± 0.09	*	9.49 ± 0.28	*
GO	8.95 ± 0.23	9.93 ± 0.05	*	10.29 ± 0.33	*
SO	5.36 ± 0.04	6.04 ± 0.08	*	9.20 ± 0.63	*
RO	3.98 ± 0.30	5.54 ± 0.38	*	9.04 ± 0.08	*
Control_2	5.44 ± 0.10	6.20 ± 0.02	*	10.24 ± 0.29	*
RD	4.94 ± 0.11	6.17 ± 0.09	*	9.73 ± 0.42	*
GOD	5.92 ± 0.09	6.68 ± 0.05	*	9.98 ± 0.45	*
S18D	5.64 ± 0.06	6.34 ± 0.08	*	9.53 ± 0.47	*
S24D	5.47 ± 0.31	6.31 ± 0.29	*	9.37 ± 0.20	*
ROD	3.38 ± 0.07	4.19 ± 0.02	*	8.92 ± 0.29	*

Values are means ± SD. The analysis was conducted in triplicates.
Significant differences between initial and final moisture content at $p < 0.05$ are shown with (*).

Table S2. Color L* values of FFSF samples at day zero, week 4 (t4), week 8 (t8) and week 12 (t12) under RTC and AC

Sample	L* Day Zero (t0)	L* RTC t4	L* RTC t8	L* RTC t12	L* AC t4	L* AC t8	L* AC t12
**Control-1**	80.42 ± 0.48 ^{d,t}	80.59 ± 0.33 ^{c,t}	80.31 ± 0.28 ^{b,t}	79.90 ± 0.10 ^{b,t}	79.76 ± 0.13 ^{b,t}	76.68 ± 0.28 ^{a,u}	70.47 ± 1.21 ^{b,v}
**GO**	69.59 ± 0.44 ^{g,t}	69.72 ± 0.36 ^{g,t}	67.94 ± 0.43 ^{g,u}	67.31 ± 0.24 ^{g,v}	63.48 ± 0.36 ^{h,w}	60.69 ± 0.82 ^{f,x}	51.66 ± 0.22 ^{h,y}
**SO**	81.53 ± 0.19 ^{c,t}	81.74 ± 0.03 ^{a,t}	81.10 ± 0.33 ^{a,t}	80.24 ± 0.25 ^{b,t}	80.81 ± 0.18 ^{a,tu}	76.93 ± 0.31 ^{a,u}	71.47 ± 0.29 ^{a,v}
**RO**	78.19 ± 0.37 ^{e,tu}	78.51 ± 0.16 ^{d,t}	78.05 ± 0.16 ^{d,u}	77.92 ± 0.29 ^{de,u}	78.13 ± 0.17 ^{c,u}	70.86 ± 0.11 ^{b,v}	60.11 ± 0.09 ^{f,w}
**Control-2**	82.55 ± 0.20 ^{b,t}	78.38 ± 0.22 ^{d,u}	78.82 ± 0.20 ^{c,u}	78.72 ± 0.48 ^{c,u}	73.55 ± 0.59 ^{f,v}	70.55 ± 0.15 ^{b,w}	68.51 ± 0.11 ^{c,x}
**RD**	81.60 ± 0.40 ^{c,t}	78.32 ± 0.27 ^{d,u}	78.07 ± 0.16 ^{d,u}	78.34 ± 0.19 ^{cd,u}	75.55 ± 0.13 ^{e,v}	62.82 ± 0.03 ^{e,w}	61.41 ± 0.17 ^{e,x}
**GOD**	80.44 ± 0.34 ^{d,t}	76.73 ± 0.21 ^{f,v}	77.14 ± 0.39 ^{e,uv}	77.47 ± 0.61 ^{e,u}	71.17 ± 0.05 ^{g,w}	67.28 ± 0.20 ^{d,x}	63.33 ± 0.50 ^{d,y}
**S18D**	84.98 ± 0.38 ^{a,t}	81.12 ± 0.13 ^{b,u}	81.03 ± 0.10 ^{a,u}	81.26 ± 0.25 ^{a,u}	76.41 ± 0.12 ^{d,v}	68.09 ± 0.17 ^{c,w}	67.82 ± 0.29 $^{c.w}$
**S24D**	80.43 ± 0.49 ^{d,t}	76.95 ± 0.04 ^{f,v}	77.57 ± 0.14 ^{de,u}	77.70 ± 0.42 ^{e,u}	75.30 ± 0.11 ^{e,w}	67.52 ± 0.11 ^{d,x}	68.19 ± 0.41 ^{c,y}
**ROD**	77.13 ± 0.72 ^{f,t}	77.75 ± 0.34 ^{e,t}	72.01 ± 0.69 ^{f,v}	70.85 ± 0.44 ^{f,w}	73.72 ± 0.25 ^{f,u}	58.06 ± 0.83 ^{g,x}	54.43 ± 0.24 ^{g,y}

Values are means ± SD. The analysis was conducted in triplicates.
$^{(a\text{-}h)}$ Means within each column followed by the same superscript letter are not significantly different ($p > 0.05$).
$^{(t\text{-}y)}$ Means within each row followed by the same letter are not significantly different ($p > 0.05$).

Table S3. Color (a*) values of FFSF samples at day zero, week 4 (t4), week 8 (t8) and week 12 (t12) under RTC and AC.

Sample	a*						
	Day Zero (t0)	RTC			AC		
		t4	t8	t12	t4	t8	t12
**Control-1**	1.62 ± 0.03 ^{g,w}	1.36 ± 0.03 ^{fg,w}	1.66 ± 0.07 ^{de,w}	1.62 ± 0.05 ^{e,w}	2.40 ± 0.18 ^{h,v}	4.09 ± 0.06 ^{g,u}	7.63 ± 0.46 ^{cd,t}
**GO**	4.48 ± 0.11 ^{b,y}	3.96 ± 0.22 ^{b,z}	4.99 ± 0.17 ^{b,x}	5.33 ± 0.16 ^{b,w}	8.55 ± 0.12 ^{b,v}	12.94 ± 0.34 ^{a,u}	14.58 ± 0.12 ^{a,t}
**SO**	0.71 ± 0.04 ^{h,w}	0.63 ± 0.01 ^{i,x}	0.77 ± 0.04 ^{g,w}	0.74 ± 0.02 ^{g,w}	1.55 ± 0.07 ^{i,v}	3.58 ± 0.02 ^{h,u}	6.55 ± 0.06 ^{d,t}
**RO**	2.84 ± 0.02 ^{d,w}	2.15 ± 0.02 ^{d,y}	2.63 ± 0.13 ^{c,x}	2.62 ± 0.09 ^{c,x}	3.39 ± 0.06 ^{fg,v}	4.85 ± 0.10 ^{f,u}	6.36 ± 0.10 ^{d,t}
**Control-2**	2.16 ± 0.04 ^{f,v}	0.87 ± 0.03 ^{h,w}	0.89 ± 0.06 ^{f,w}	0.83 ± 0.04 ^{g,w}	4.60 ± 0.15 ^{e,u}	9.39 ± 0.24 ^{b,t}	9.31 ± 0.17 ^{b,t}
**RD**	3.25 ± 0.11 ^{c,w}	2.63 ± 0.15 ^{c,x}	1.88 ± 0.01 ^{d,y}	1.75 ± 0.05 ^{d,y}	6.36 ± 0.10 ^{c,v}	8.38 ± 0.07 ^{c,u}	9.92 ± 0.09 ^{b,t}
**GOD**	2.94 ± 0.04 ^{d,w}	1.64 ± 0.03 ^{e,x}	1.61 ± 0.09 ^{de,x}	1.57 ± 0.10 ^{e,x}	5.04 ± 0.03 ^{d,v}	7.12 ± 0.12 ^{e,u}	8.71 ± 0.12 ^{bc,t}
**S18D**	2.54 ± 0.07 ^{e,w}	1.26 ± 0.01 ^{g,x}	1.32 ± 0.01 ^{ef,x}	1.28 ± 0.05 ^{f,x}	3.30 ± 0.06 ^{g,v}	7.65 ± 0.13 ^{d,u}	8.20 ± 0.14 ^{bc,t}
**S24D**	2.68 ± 0.07 ^{e,w}	1.41 ± 0.02 ^{fg,x}	1.31 ± 0.01 ^{ef,y}	1.36 ± 0.04 ^{f,xy}	3.49 ± 0.01 ^{f,v}	7.07 ± 0.05 ^{e,u}	7.79 ± 0.06 ^{bc,t}
**ROD**	8.96 ± 0.23 ^{a,w}	7.82 ± 0.02 ^{a,x}	6.59 ± 0.24 ^{a,y}	6.34 ± 0.04 ^{a,y}	9.25 ± 0.05 ^{a,v}	9.63 ± 0.17 ^{b,u}	10.27 ± 0.11 ^{bc,t}

Values are means ± SD. The analysis was conducted in triplicates.
[(a-i)] Means within each column followed by the same letter are not significantly different ($p > 0.05$).
[(t-z)] Means within each row followed by the same letter are not significantly different ($p > 0.05$).

Table S4. Color (b)* values of FFSF samples at day zero, week 4 (t4), week 8 (t8) and week 12 (t12) under RTC and AC.

Sample	b*						
	Day Zero (t0)	RTC			AC		
		t4	t8	t12	t4	t8	t12
**Control-1**	21.80 ± 0.18 ^{e,w}	19.34 ± 0.68 ^{e,x}	23.36 ± 0.25 ^{c,v}	22.89 ± 0.19 ^{c,vw}	23.19 ± 0.97 ^{e,v}	26.85 ± 0.74 ^{e,u}	31.47 ± 0.82 ^{b,t}
**GO**	27.07 ± 0.18 ^{b,x}	25.93 ± 0.59 ^{a,y}	30.24 ± 0.70 ^{a,w}	31.18 ± 0.53 ^{a,vw}	31.44 ± 0.37 ^{a,v}	43.86 ± 0.60 ^{a,u}	47.91 ± 0.63 ^{a,t}
**SO**	18.76 ± 0.05 ^{g,wx}	17.30 ± 0.06 ^{f,x}	20.42 ± 0.41 ^{d,vw}	19.40 ± 0.46 ^{e,wx}	21.26 ± 0.12 ^{g,v}	26.00 ± 0.19 ^{e,u}	28.81 ± 0.29 ^{c,t}
**RO**	23.80 ± 0.13 ^{d,x}	21.46 ± 0.28 ^{c,y}	24.70 ± 0.97 ^{b,w}	25.53 ± 0.27 ^{b,v}	25.12 ± 0.30 ^{c,vw}	28.96 ± 0.12 ^{c,u}	31.28 ± 0.40 ^{b,t}
**Control-2**	24.82 ± 0.27 ^{c,u}	20.27 ± 0.32 ^{d,wx}	20.42 ± 0.21 ^{d,w}	19.55 ± 0.43 ^{e,x}	23.43 ± 0.29 ^{de,v}	29.18 ± 0.96 ^{c,t}	29.35 ± 0.38 ^{c,t}
**RD**	23.43 ± 0.30 ^{d,x}	19.60 ± 0.70 ^{de,w}	18.55 ± 0.08 ^{e,y}	17.51 ± 0.17 ^{f,z}	25.56 ± 0.14 ^{c,v}	27.81 ± 0.21 ^{d,u}	29.31 ± 0.23 ^{c,t}
**GOD**	22.03 ± 0.22 ^{e,w}	17.96 ± 0.11 ^{f,x}	17.63 ± 0.11 ^{f,y}	16.34 ± 0.16 ^{g,z}	23.92 ± 0.02 ^{d,u}	26.75 ± 0.34 ^{e,t}	23.58 ± 0.17 ^{e,v}
**S18D**	20.72 ± 0.31 ^{f,u}	15.95 ± 0.06 ^{f,v}	15.99 ± 0.09 ^{f,v}	15.78 ± 0.14 ^{h,v}	21.10 ± 0.38 ^{g,u}	26.81 ± 0.67 ^{e,t}	26.80 ± 0.20 ^{d,t}
**S24D**	22.10 ± 0.40 ^{e,w}	17.36 ± 0.02 ^{f,x}	17.26 ± 0.22 ^{f,x}	15.70 ± 0.06 ^{h,y}	22.49 ± 0.02 ^{f,v}	28.07 ± 0.23 ^{d,t}	27.48 ± 0.06 ^{d,u}
**ROD**	27.51 ± 0.12 ^{a,v}	24.55 ± 0.46 ^{b,w}	23.80 ± 0.68 ^{c,x}	22.38 ± 0.14 ^{d,y}	28.34 ± 0.05 ^{b,u}	30.38 ± 0.17 ^{b,t}	30.88 ± 0.18 ^{b,t}

Values are means ± SD. The analysis was conducted in triplicates.
[(a-h)] Means within each column followed by the same letter are not significantly different ($p > 0.05$).
[(t-z)] Means within each row followed by the same letter are not significantly different ($p > 0.05$).

Table S5. Chroma values of FFSF samples at day zero, week 4 (t4), week 8 (t8) and week 12 (t12) under RTC and AC.

Sample	CHR						
	Day Zero (t0)	RTC			AC		
		t4	t8	t12	t4	t8	t12
**Control-1**	21.86 ± 0.18 ^{e,w}	19.38 ± 0.68 ^{d,x}	23.42 ± 0.25 ^{c,v}	22.95 ± 0.19 ^{c,vw}	23.31 ± 0.98 ^{fg,v}	27.16 ± 0.73 ^{e,u}	32.38 ± 0.90 ^{b,t}
**GO**	27.44 ± 0.20 ^{b,x}	26.23 ± 0.62 ^{a,y}	30.65 ± 0.72 ^{a,w}	31.63 ± 0.55 ^{a,vw}	32.58 ± 0.39 ^{a,v}	45.73 ± 0.66 ^{a,u}	50.08 ± 0.63 ^{a,t}
**SO**	18.77 ± 0.05 ^{g,y}	17.31 ± 0.06 ^{f,z}	20.43 ± 0.41 ^{d,w}	19.41 ± 0.46 ^{d,x}	21.32 ± 0.12 ^{h,v}	26.25 ± 0.19 ^{f,u}	29.55 ± 0.27 ^{d,t}
**RO**	23.97 ± 0.12 ^{d,x}	21.57 ± 0.29 ^{b,y}	24.84 ± 0.98 ^{b,w}	25.67 ± 0.26 ^{b,vw}	25.34 ± 0.30 ^{d,v}	29.36 ± 0.11 ^{d,u}	31.92 ± 0.38 ^{b,t}
**Control-2**	24.91 ± 0.27 ^{c,u}	20.29 ± 0.32 ^{c,wx}	20.44 ± 0.21 ^{d,w}	19.57 ± 0.43 ^{d,x}	23.87 ± 0.28 ^{ef,v}	30.66 ± 0.98 ^{c,t}	30.79 ± 0.40 ^{c,t}
**RD**	23.65 ± 0.31 ^{d,w}	19.77 ± 0.71 ^{cd,x}	18.65 ± 0.08 ^{e,y}	17.59 ± 0.17 ^{e,z}	26.34 ± 0.12 ^{c,v}	29.04 ± 0.20 ^{d,u}	30.95 ± 0.23 ^{c,t}
**GOD**	22.23 ± 0.22 ^{e,w}	18.04 ± 0.11 ^{e,x}	17.70 ± 0.11 ^{f,y}	16.42 ± 0.16 ^{f,z}	24.44 ± 0.01 ^{e,v}	27.69 ± 0.31 ^{e,u}	25.14 ± 0.12 ^{f,t}
**S18D**	20.87 ± 0.31 ^{f,u}	16.00 ± 0.06 ^{g,v}	16.05 ± 0.09 ^{g,v}	15.83 ± 0.14 ^{g,v}	21.36 ± 0.38 ^{h,u}	27.88 ± 0.67 ^{e,t}	28.03 ± 0.15 ^{e,t}
**S24D**	22.26 ± 0.41 ^{e,w}	17.41 ± 0.02 ^{ef,x}	17.31 ± 0.22 ^{f,x}	15.76 ± 0.07 ^{g,y}	22.76 ± 0.01 ^{g,v}	28.95 ± 0.21 $^{d.t}$	28.57 ± 0.07 ^{e,u}
**ROD**	28.93 ± 0.05 ^{a,w}	25.76 ± 0.43 ^{a,x}	24.70 ± 0.72 ^{b,y}	23.26 ± 0.12 ^{c,z}	29.81 ± 0.04 ^{b,v}	31.87 ± 0.16 ^{b,u}	32.55 ± 0.20 ^{b,t}

Values are means ± SD. The analysis was conducted in triplicates.

$^{(a-h)}$ Means within each column followed by the same letter are not significantly different ($p > 0.05$).

$^{(t-z)}$ Means within each row followed by the same letter are not significantly different ($p > 0.05$).

Table S6. Hue angle values of FFSF samples at day zero, week 4 (t4), week 8 (t8) and week 12 (t12) under RTC and AC.

Sample	HUE						
	Day Zero (t0)	RTC			AC		
		t4	t8	t12	t4	t8	t12
**Control-1**	85.74 ± 0.04 ^{b,t}	85.97 ± 0.11 ^{c,t}	85.93 ± 0.12 ^{c,t}	85.95 ± 0.11 ^{b,t}	84.09 ± 0.19 ^{b,u}	81.34 ± 0.31 ^{b,v}	76.39 ± 0.46 ^{c,w}
**GO**	80.60 ± 0.17 ^{f,uv}	81.32 ± 0.29 ^{h,t}	80.64 ± 0.18 ^{i,u}	80.30 ± 0.12 ^{g,v}	74.78 ± 0.04 ^{h,w}	73.57 ± 0.23 ^{g,x}	73.07 ± 0.17 ^{e,y}
**SO**	87.84 ± 0.11 ^{a,t}	87.93 ± 0.04 ^{a,t}	87.84 ± 0.08 ^{a,t}	87.81 ± 0.10 ^{a,t}	85.82 ± 0.16 ^{a,u}	82.17 ± 0.09 ^{a,v}	77.19 ± 0.23 ^{b,w}
**RO**	83.19 ± 0.07 ^{d,v}	84.28 ± 0.03 ^{f,t}	83.93 ± 0.06 ^{h,u}	84.14 ± 0.25 ^{f,tu}	82.31 ± 0.07 ^{c,w}	80.49 ± 0.21 ^{c,x}	78.50 ± 0.31 ^{a,y}
**Control-2**	85.03 ± 0.05 ^{c,u}	87.55 ± 0.08 ^{b,t}	87.50 ± 0.13 ^{b,t}	87.57 ± 0.09 ^{a,t}	78.90 ± 0.38 ^{e,v}	72.17 ± 0.12 ^{h,w}	72.40 ± 0.19 ^{f,w}
**RD**	82.10 ± 0.16 ^{e,u}	82.37 ± 0.30 ^{g,u}	84.21 ± 0.05 ^{g,t}	84.28 ± 0.19 ^{ef,t}	76.03 ± 0.25 ^{g,v}	73.23 ± 0.20 ^{g,w}	71.30 ± 0.15 ^{g,x}
**GOD**	82.40 ± 0.18 ^{e,u}	84.79 ± 0.11 ^{e,t}	84.79 ± 0.28 ^{f,t}	84.51 ± 0.33 ^{e,t}	78.10 ± 0.07 ^{f,v}	75.09 ± 0.39 ^{e,w}	69.73 ± 0.39 ^{h,x}
**S18D**	83.02 ± 0.10 ^{d,u}	85.48 ± 0.04 ^{d,t}	85.27 ± 0.03 ^{e,t}	85.38 ± 0.15 ^{c,t}	81.10 ± 0.24 ^{d,v}	74.08 ± 0.13 ^{f,w}	72.99 ± 0.39 ^{e,x}
**S24D**	83.09 ± 0.06 ^{d,w}	85.34 ± 0.05 ^{d,u}	85.66 ± 0.03 ^{d,t}	85.05 ± 0.13 ^{d,v}	81.19 ± 0.02 ^{d,x}	75.87 ± 0.21 ^{d,y}	74.18 ± 0.09 ^{d,z}
**ROD**	71.97 ± 0.51 ^{g,uv}	72.33 ± 0.35 ^{I,u}	74.52 ± 0.16 ^{j,t}	74.18 ± 0.19 ^{h,t}	71.93 ± 0.12 ^{I,uv}	72.42 ± 0.32 ^{h,u}	71.61 ± 0.10 ^{g,v}

Values are mean ± SD. The analysis was conducted in triplicates.

$^{(a-h)}$ Means within each column followed by the same letter are not significantly different ($p > 0.05$).

$^{(t-z)}$ Means within each row followed by the same letter are not significantly different ($p > 0.05$).

Table S7. Total color change (ΔE1) between control and pretreated samples on day zero (t0) and total color change (ΔE2) in samples kept under RTC and AC at the end of twelve-week storage time.

Sample	ΔE1	ΔE2	
		RTC	AC
Control-1	-	1.21 ± 0.21 [d]	15.12 ± 1.50 [ef]
GO	12.38 ± 0.48 [a]	4.77 ± 0.59 [c]	29.29 ± 0.61 [a]
SO	3.36 ± 0.11 [d]	1.48 ± 0.28 [d]	15.38 ± 0.23 [ef]
RO	3.24 ± 0.32 [d]	1.78 ± 0.29 [d]	19.88 ± 0.21 [c]
Control-2	-	6.67 ± 0.18 [b]	16.40 ± 0.16 [de]
RD	2.05 ± 0.03 [e]	6.93 ± 0.17 [b]	22.06 ± 0.22 [b]
GOD	3.59 ± 0.27 [d]	6.58 ± 0.18 [b]	18.12 ± 0.50 [cd]
S18D	4.79 ± 0.45 [c]	6.31 ± 0.11 [b]	19.07 ± 0.27 [c]
S24D	3.52 ± 0.08 [d]	7.09 ± 0.11 [b]	14.32 ± 0.38 [f]
ROD	9.11 ± 0.57 [b]	8.52 ± 0.39 [a]	22.99 ± 0.22 [b]

Values are mean ± SD. The analysis was conducted in triplicates.

[(a-f)] Means within each column followed by the same letter are not significantly different ($p > 0.05$).

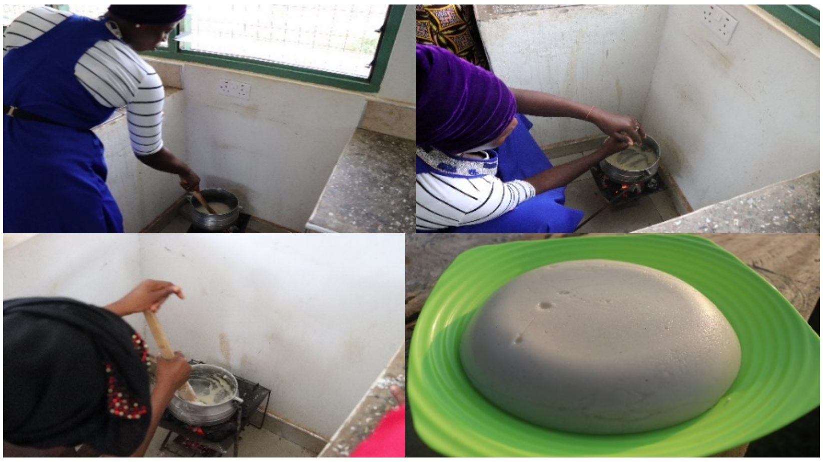

Figure S1. Preparation of Tuo Zaafi by women for sensory evaluation.

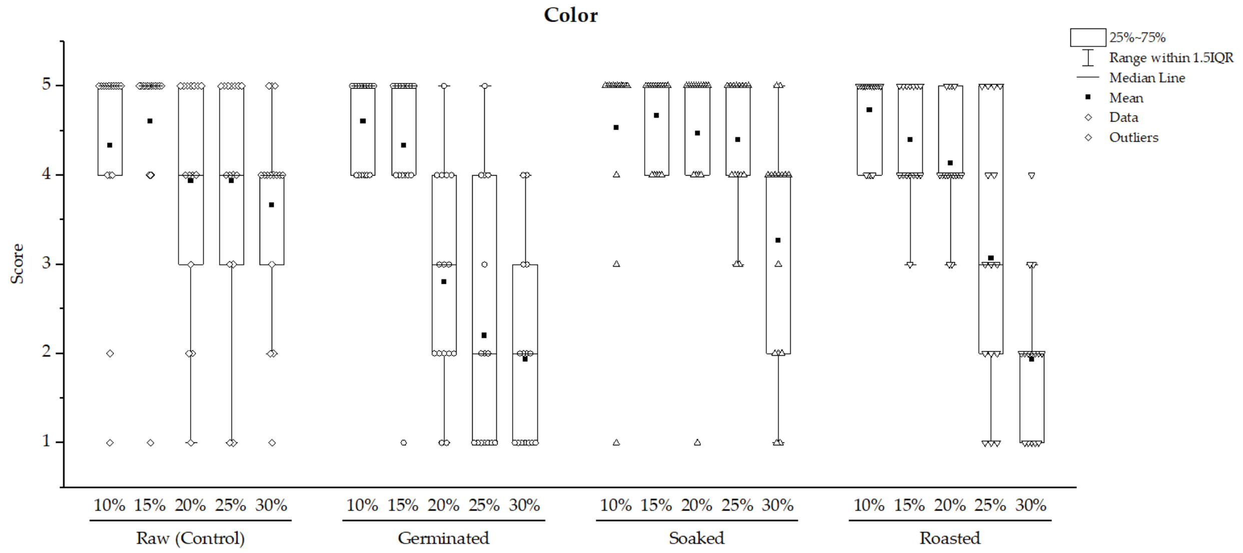

Figure S2. Sensory evaluation scores (color) of pretreated FFSF-enriched Tuo-Zaafi by women (n=15) in Ghana.

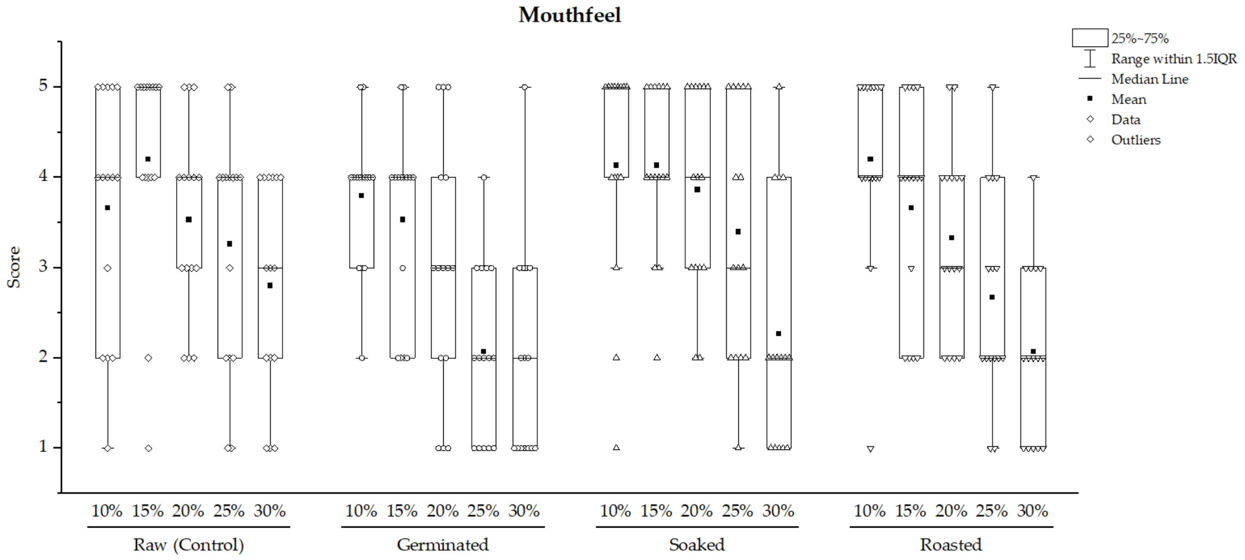

Figure S3. Sensory evaluation scores (mouthfeel) of pretreated FFSF-enriched Tuo-Zaafi by women (n=15) in Ghana.

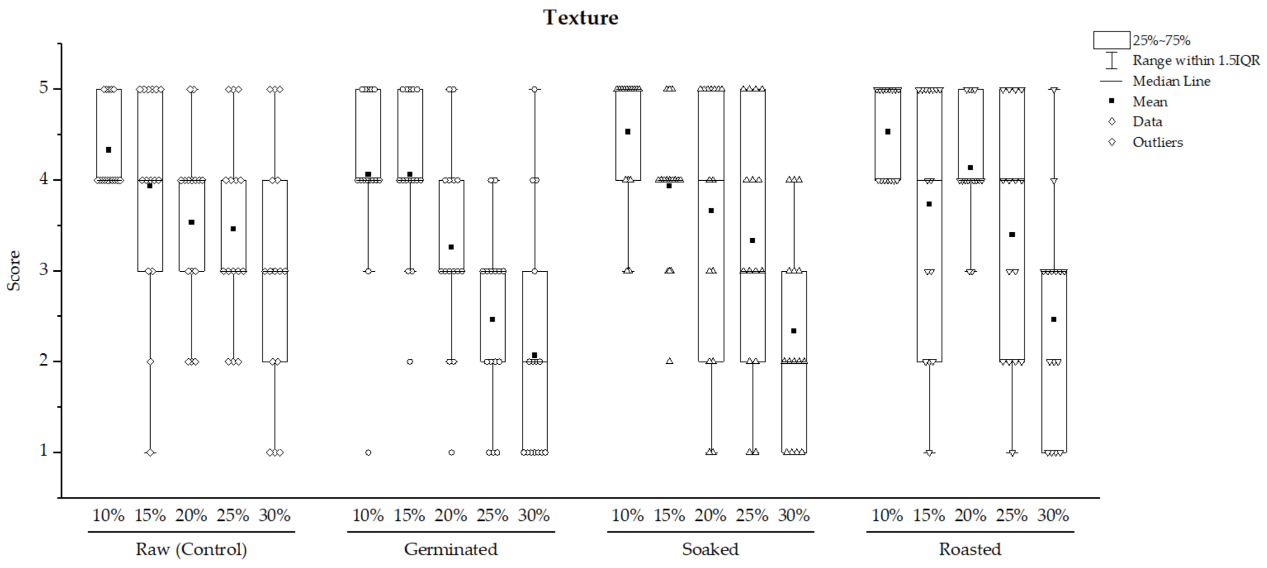

Figure S4. Sensory evaluation scores (texture) of pretreated FFSF-enriched Tuo-Zaafi by women (n=15) in Ghana.

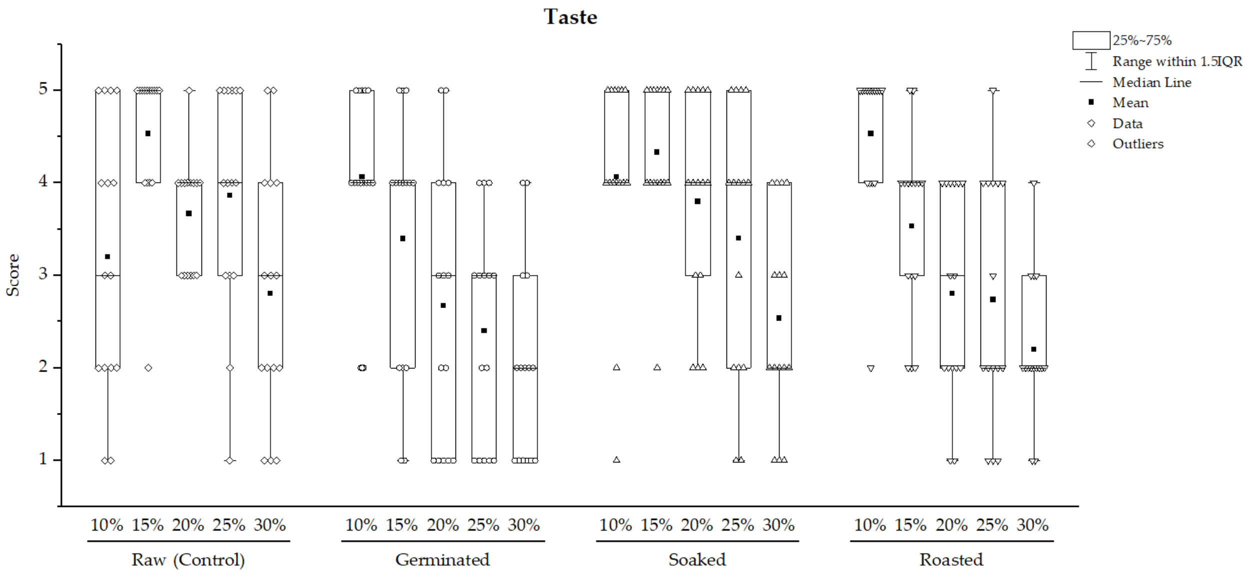

Figure S5. Sensory evaluation scores (taste) of pretreated FFSF-enriched Tuo-Zaafi by women (n=15) in Ghana.

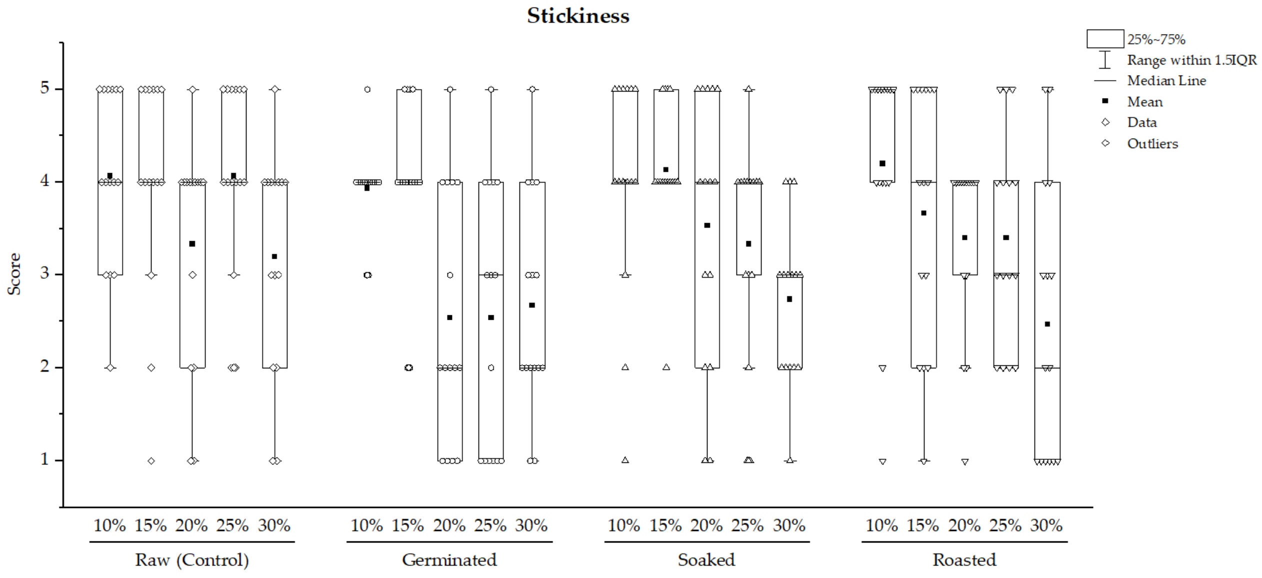

Figure S6. Sensory evaluation scores (stickiness) of pretreated FFSF-enriched Tuo-Zaafi by women (n=15) in Ghana.

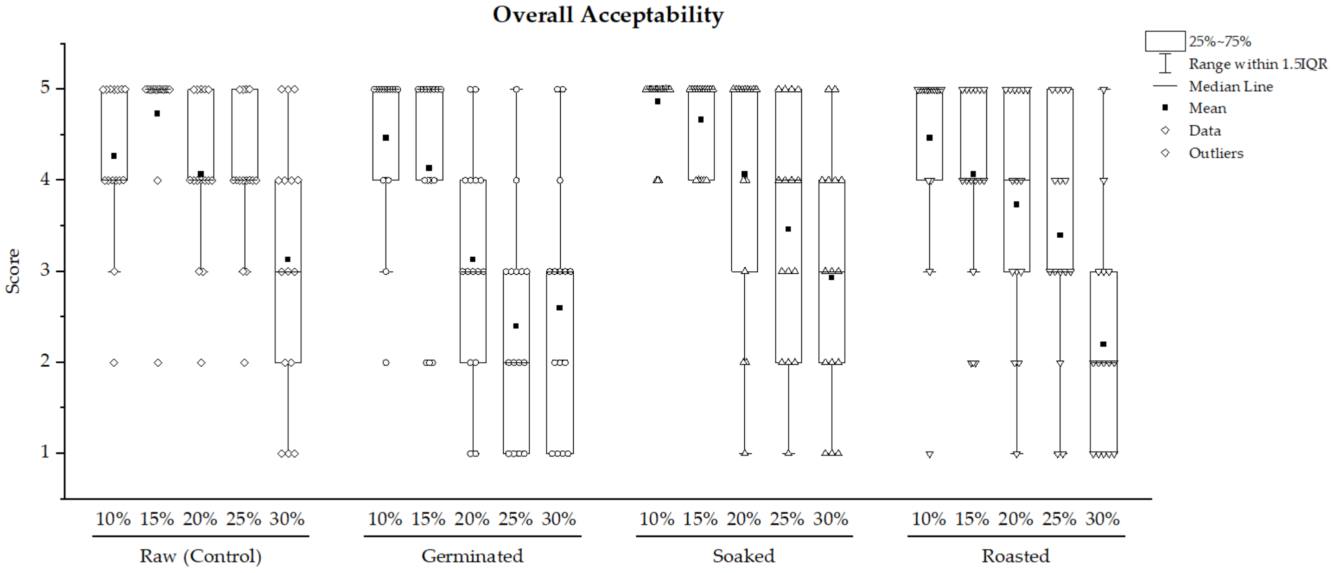

Figure S7. Sensory evaluation scores (overall acceptability) of pretreated FFSF-enriched Tuo-Zaafi by women (n=15) in Ghana.

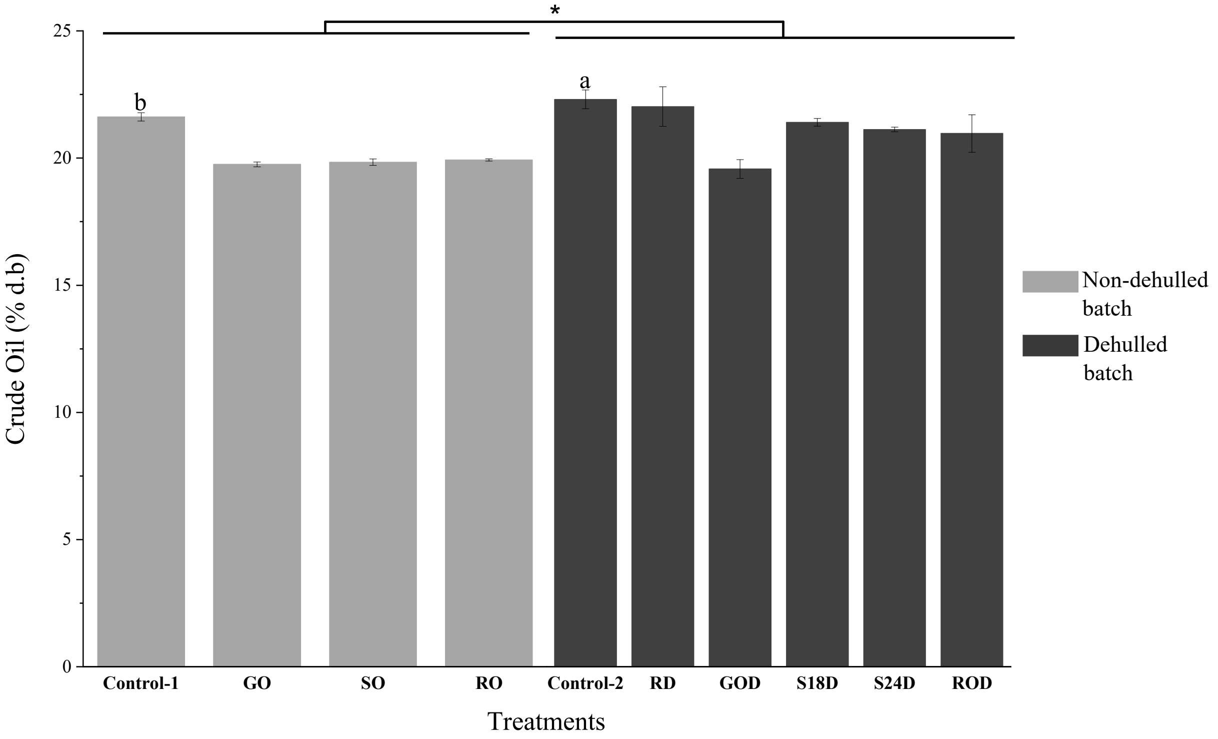

Figure S8. Crude oil yields of FFSF samples measured on day zero before storage. Differences between the group of dehulled samples and non-dehulled batches is shown with (*) at $p < 0.05$. Different letters (a-b) denote significant difference between Control-1 and Control-2 samples ($p < 0.05$).
